# Effect of Comorbidities on Inflammatory Bowel Disease-Related Colorectal Cancer: A Nationwide Inpatient Review

**DOI:** 10.7759/cureus.27599

**Published:** 2022-08-02

**Authors:** Arnold N Forlemu, Raissa Nana Sede Mbakop, Shehroz Aslam, Zaid Ansari, Indu Srinivasan, Keng-Yu Chuang

**Affiliations:** 1 Internal Medicine/Gastroenterology and Hepatology, University of Nebraska Medical Center, Omaha, USA; 2 Gastroenterology, Universite De Rennes 1, Rennes, FRA; 3 Internal Medicine, Piedmont Athens Regional, Athens, USA; 4 Internal Medicine/Gastroenterology, Creighton University School of Medicine, Phoenix, USA

**Keywords:** cancer surveillance, colorectal neoplasia, crohn’s disease, ulcerative colitis, liver disease, comorbidities

## Abstract

Introduction

The risk of inflammatory bowel disease-associated colorectal cancer (IBD-CRC) is known to increase with primary sclerosing cholangitis (PSC) and a family history of CRC. However, the impact of comorbidities such as liver disease, obesity, diabetes, chronic lung, heart, and renal disease, and psychiatric illness on the risk of IBD-CRC remains unclear. We evaluated the effect of these comorbidities on the risk of IBD-CRC.

Methods

A retrospective review from 2009 to 2014 was conducted using the National Inpatient Sample data for adults 18 years and older. Patients with IBD (360,892), of whom 2,831 had CRC were identified using the International Classification of Diseases, Ninth Revision codes (ICD-9). Data on comorbidities were also obtained. Adjusted odds ratios (aOR) and confidence intervals (CI) were computed via logistic regression to evaluate the effect of comorbidities on the risk of IBD-CRC; the p-value was set at <0.05.

Results

The mean age of IBD patients in this study was 52.36±0.03. A majority of the patients with IBD-CRC were white and were significantly older compared to those without cancer (60 vs 52 years, p<0.05). The risk of colon cancer in IBD was increased by having a non-cholestatic liver disease (aOR 1.51, CI 1.23-1.86, p<0.01). Also, patients younger than 50 years with liver disease were at an increased risk of IBD-associated colon cancer in comparison to older patients (aOR 1.83 vs 1.34, p<0.05). Notably, diabetes, chronic pulmonary disease, renal failure, psychiatric illnesses, and rheumatoid diseases, were inversely associated with the risk of IBD-CRC (p<0.05). After stratifying by IBD subtypes, non-cholestatic liver disease was still independently associated with a higher risk for colon cancer in patients with ulcerative colitis or Crohn's disease (ulcerative colitis: aOR 1.43, CI 1.08-1.89; Crohn's disease: aOR 1.46, CI 1.10-2.00).

Conclusions

Patients with IBD who have non-cholestatic liver disease might have a higher risk for colon cancer, even at a younger age. These patients may require close colon cancer surveillance.

## Introduction

Inflammatory bowel disease-associated colorectal cancer (IBD-CRC) is a serious complication of inflammatory bowel disease (IBD); accounting for about 2% of all colorectal cancer (CRC), and 15% of all-cause mortality among IBD patients [1]. It is frequently diagnosed at an advanced cancer stage as early IBD-CRC detection has remained a clinical challenge [1]. The risk of IBD-CRC is known to increase with a family history of CRC, primary sclerosing cholangitis (PSC), disease duration, disease extension, and active intestinal inflammation [2,3]. However, the effect of comorbidities such as diabetes, obesity, chronic lung diseases, and liver disease on the risk of IBD-CRC remains unclear. A recent study in Canada by Loo et al. found respiratory diseases, chronic kidney disease, and diabetes to be associated with cancer in patients with IBD [4]. The direct association between frequent comorbidities and IBD-CRC risk in the US remains to be established. Given that IBD-CRC has a worse prognosis than sporadic CRC [1], early detection of IBD-CRC is paramount. It is necessary to identify comorbidities and risk factors that may raise awareness to warrant closer surveillance of IBD-CRC to help decrease morbidity and mortality associated with this condition. The purpose of this study was to evaluate the effect of comorbidities on the risk of IBD-CRC.

## Materials and methods

A retrospective review from 2009 to 2014 was conducted using the National Inpatient Sample (NIS) data to identify IBD patients diagnosed with CRC. The NIS is the largest publicly available all-payer inpatient database in the US with more than seven million hospital stays each year [5], as a part of the Healthcare Cost and Utilization Project (HCUP). It is designed to be nationally representative and captures a significant number of community hospitals in the US. The HCUP database contains de-identified data on nationwide hospital admissions including demographic information, clinical data, comorbidities, discharge diagnoses, procedures, outcomes, and hospitalization costs. The NIS database list patients with a primary discharge diagnosis and up to 29 secondary discharge diagnoses and 15 procedure codes. It lacks information on clinical details such as clinical stages of a disease, laboratory, and pharmacy data [5]. This study was approved by our institutional review board (IRB) and deemed exempt from IRB review due to the de-identified nature of HCUP data (IRB number 2021-031).

Study sample

Patients with IBD were identified using the International Classification of Diseases, Ninth Revision, Clinical Modification (ICD-9-CM) codes for ulcerative colitis and Crohn’s disease (556.x and 555.x), respectively. Patients with ulcerative colitis (UC) and Crohn’s disease (CD) were then grouped to constitute IBD patients. Likewise, patients with CRC within the IBD patient population were identified with ICD-9-CM codes 153.x. and 154.x. (Table 1).

**Table 1:** ICD-9-CM diagnostic codes and AHRQ comorbidity groups ICD-9-CM: International Classification of Diseases, Ninth Revision, Clinical Modification codes, AHRQ: Agency for Healthcare Research and Quality

Description	ICD-9-CM code/AHRQ comorbidity groups
Ulcerative colitis (556.x)	556.0, 556.1, 556.2, 556.3, 556.4, 556.5, 556.6, 556.8, 556.9
Crohn’s disease (555.x)	555.0, 555.1, 555.2, 555.9
Colorectal cancer (153.x and 154.x)	153.0, 153.1, 153.2, 153.3, 153.4, 153.6, 153.7, 153.8, 153.9, 154.0, 154.1
Dyslipidemia (272.x)	272.0, 272.1, 272.2, 272.3, 272.4
Hypothyroidism	243-244.2, 244.8, 244.9
Obesity	278.0, 278.00, 278.01, 278.03, 649.10-649.14, 793.91, V85.30-V85.39, V85.41-V85.45, V85.54
Liver disease	Chronic non-cholestatic liver disease: hepatitis (B, C, alcohol, fatty liver), liver cirrhosis
Pulmonary embolism and complications	Pulmonary embolism with and without infarcts (septic, saddle, iatrogenic, chronic), pulmonary hypertension, chronic pulmonary heart disease
Diabetes	Uncomplicated and complicated diabetes
Renal failure	Chronic kidney disease (III, IV, V, endstage), renal dialysis
Hypertension	Uncomplicated and complicated hypertension
Chronic pulmonary disease	Bronchitis/emphysema, chronic pulmonary obstructive disease, asthma, bronchiectasis, interstitial lung disease
Congestive heart failure	Congestive heart failure (rheumatic, systolic, diastolic, combined, acute, chronic, acute on chronic)
Rheumatoid diseases	Scleroderma, systemic lupus erythematosus, sicca syndrome, rheumatoid arthritis, polymyositis, dermatomyositis, ankylosing spondylitis, polymyalgia rheumatica, connective tissue disorders
Psychoses	Schizophrenia, manic disorders, psychosis, paranoid
Depression	Depressive disorders excluding ones with psychoses

We analyzed CRC as a whole, and as rectal and colon cancers individually to reflect some evidence suggesting that sporadic colon and rectal cancers may behave differently in terms of carcinogenesis, pathology, surgical topography, treatment modalities, and outcomes [6]; and these cancers may also behave differently in inflammation-related CRC. Rectosigmoid junction cancers were excluded during the individual analyses of colon and rectal cancers to minimize misclassification. All participants included in this study were aged 18 years or older.

Covariates

Demographic information including age, gender, race/ethnicity, primary payer information (Medicare, Medicaid, private insurance, self-pay, other insurance), teaching status, and hospital location (rural vs urban), were obtained. Data on obesity, diabetes, renal failure, chronic respiratory disease, liver disease, congestive heart failure (CHF), pulmonary embolism (PE) and complications, dyslipidemia, depression, hypothyroidism, psychoses, and rheumatoid diseases were also obtained. Comorbidity measures in HCUP were assigned using the Elixhauser Agency for Healthcare Research and Quality (AHRQ) comorbidity software (Healthcare Cost and Utilization Project (HCUP), Rockville, MD, USA) which identifies coexisting medical conditions not directly related to the principal diagnosis and therefore likely to have originated prior to the hospital stay [7]. Each comorbidity may comprise more than one etiology of disease in an organ system as seen above in Table 1. We excluded 5,395 cases of CHF, hypertension, and renal failure to minimize misclassification because some of their ICD codes assigned by the AHRQ comorbidity software were shared. Likewise, we excluded patients with PSC, cholangitis or cholestatic disease, transplant liver disease, and biliary cirrhosis from the analysis to minimize confounding. Primary sclerosing cholangitis is a known risk factor for IBD-CRC, and ICD-9-codes for PSC include codes for other cholangitic and biliary conditions, hence they were excluded. Also, PSC is one of the primary reasons for liver transplantation in IBD patients, hence liver transplant patients with IBD likely also had PSC. Patients with hepatopulmonary syndrome and IBD were excluded due to very few cases.

Outcomes of interest

We analyzed the following outcomes: 1) prevalence of comorbidities associated with CRC, colon cancer (CC), and rectal cancer (RC) in IBD patients; 2) adjusted odds ratios (OR) of comorbidities associated with CRC, CC, and RC in IBD patients; 3) adjusted ORs of comorbidities associated with CRC, CC, and RC in IBD stratified by UC and CD patients.

Statistical analysis

Data were analyzed using Statistical Package for Social Sciences (SPSS) version 24.0 (IBM Corp., Armonk, NY, USA). The data are presented as weighted frequencies for categorical variables and as mean ± standard error for continuous variables. Chi-square statistics were used to compare proportions for categorical variables and Student's t-test was used to compare means for continuous variables. A univariate analysis was first conducted for the main effects to identify factors that were potentially associated with CRC in IBD patients. Logistic regression was then used to obtain adjusted estimates for comorbidities associated with CRC, CC, and RC in patients with IBD. Statistical significance was set at a two-tailed p<0.05 for all tests. Estimates were presented as odds ratios (ORs) and 95% confidence intervals (CI).

## Results

Baseline characteristics

The number of inpatient admissions with a principal diagnosis of UC and CD totaled 360,892, of which 2,831 cases had IBD-CRC, 1,884 cases had IBD-CC, and 723 had IBD-RC. Of the IBD-CRC cases, 1,426 were identified in UC patients and 1,405 in CD patients. The mean age of IBD patients in this study was 52.36±0.03 (Table 2).

**Table 2:** Baseline characteristics of IBD patients PE: Pulmonary embolism; Other race: Includes Asian/Pacific Islanders, Native American, and mixed races; mean±SE: mean±standard error; IBD: Inflammatory bowel disease

Characteristic	IBD, N=360,892	Ulcerative colitis, N=132,035 (36.6%)	Crohn’s disease, N=228,857 (63.8%)
Age (in years) Mean±SE	52.36±0.03	55.54±0.06	50.52±18.0.04
Age (in years)			
18-35	86,813 (24.1%)	27,519 (20.8%)	59,294 (25.9%)
35-50	82,139 (22.8%)	25,046 (19.0%)	57,093 (24.9%)
51-65	90,701 (25.1%)	32,673 (24.7%)	58,028 (25.4%)
>65	101,239 (28.1%)	46,797 (35.4%)	54,442 (23.8%)
Sex			
Women	206,384 (57.2%)	71,079 (53.8%)	135,305 (59.1%)
Men	154,396 (42.8%)	60,898 (46.1%)	93,498 (40.9%)
Race			
White	262,474 (72.9%)	94,916 (71.9%)	167,558 (73.2%)
Black	34,645 (9.6%)	11,173 (8.5%)	23,472 (10.3%)
Hispanic	17,591 (4.9%)	8,814 (6.7%)	8,777 (3.8%)
Asian/Pacific islander	3269 (0.9%)	1,715 (1.3%)	1,554 (0.7%)
Native American	1334 (0.4%)	473 (0.4%)	861 (0.4%)
Other	7872 (2.2%)	3,399 (2.6%)	4,473 (2.0%)
Payment method			
Medicare	141,066 (39.1%)	54,323 (41.1%)	86,743 (37.9%)
Medicaid	43,394 (12.0%)	12,718 (9.6%)	30,676 (13.4%)
Private insurance	143,700 (39.8%)	53,631 (40.6%)	90,077 (39.4%)
Self-pay	18,352 (5.1%)	6,015 (4.6%)	12,337 (5.4%)
Other insurance	2064 (0.6%)	673 (0.5%)	1,391 (0.6%)
Hospital type			
Urban	101,524 (28.1%)	38,317 (29.0%)	63,207 (27.6%)
Rural	19,245 (5.3%)	6,476 (4.9%)	12,769 (5.6%)
Teaching status			
Non-teaching hospital	81	38	43
Teaching hospital	34	16	18
Colorectal cancer	2831 (0.8%)	1,426 (1.1%)	1,405 (0.6%)
Colon cancer	1884 (0.5%)	933 (0.7%)	951 (0.4%)
Rectal cancer	723 (0.2%)	365 (0.3%)	358 (0.2%)
Rheumatoid diseases	107,028 (4.7%)	5,622 (4.3%)	11,406 (5.0%)
Congestive heart failure	19,770 (5.5%)	8,622 (6.5%)	11,148 (4.9%)
Chronic pulmonary disease	61,911 (17.2%)	21,985 (16.7%)	39,926 (17.4%)
Diabetes	55,541 (5.2%)	23,391 (17.7%)	32,150 (14.0%)
Hypertension	134,859 (37.4%)	54,037 (40.9%)	80,822 (35.3%)
Hypothyroidism	36,822 (10.2%)	14,637 (11.1%)	22,185 (9.7%)
Chronic liver disease	14,411 (4.0%)	6,251 (4.7%)	8,160 (3.6%)
Obesity	29,535 (8.2%)	11,290 (8.6%)	18,245 (8.0%)
Chronic PE	6345 (1.8%)	2,852 (2.2%)	3,493 (1.5%)
Renal failure	29,506 (8.2%)	11,416 (8.6%)	18,090 (7.9%)
Dyslipidemia	68,380 (18.9%)	30,951 (23.4%)	37,429 (16.4%)
Depression	54,297 (15.0%)	17,583 (13.3%)	36,714 (16.0%)
Psychoses	18,932 (5.2%)	5,863 (4.4%)	13,069 (5.7%)

Risk factors and comorbidities in patients with IBD-CRC compared to IBD non-CRC patients

The prevalence of IBD-CRC was higher in men compared to women. Likewise, the majority of IBD-CRC patients were white and were significantly older compared to those without cancer (60 vs 52 years, p<0.05). Patients with IBD-CC had higher proportions of liver disease (5.6% vs 4.0%), and hypertension (40.8% vs 37.4%) compared to IBD non-CC patients, p<0.01 (Table 3).

**Table 3:** Prevalence of comorbidities in IBD-CRC, IBD-CC, IBD-RC patients PE: Pulmonary embolism; Other race: Includes Asian/Pacific Islanders, Native American, and mixed races; IBD: inflammatory bowel disease; CRC: colorectal cancer; CC: colon cancer; RC: rectal cancer; a: P<0.05; b: P<0.01

Parameter	IBD (%)					
Cancer types	CRC	Non-CRC	CC	Non-CC	RC	Non-RC
Age (years) Mean±SE	60.47±0.03	52.29±0.29^*^	61.37±0.37	52.31±0.03^*^	58.80±0.53	52.34±0.03^a^
Age (years)^a^						
18 to 35	7.3	24.2	7.9	24.1	5.7	24.1
36 to 50	19.7	22.8	18.0	22.8	23.1	22.8
51 to 65	34.4	25.1	31.5	25.1	40.1	25.1
>65	38.5	28.0	42.5	28.0	31.1	28.0
Sex^a^						
Female	43.1	57.3	45.4	57.3	38.9	57.2
Male	56.9	42.7	54.6	42.7	61.1	42.8
Race						
White	82.1	80.2	82.8	80.2	81.0	80.2
Black	7.2	10.6^a^	6.6	10.6^a^	9.3	10.6
Hispanic	6.0	5.4	6.0	5.4	5.7	5.4
Other	4.7	3.8	4.6	3.8	4.0	3.8
Payment methods^a^						
Medicare	44.7	40.4	46.8	40.4	41.5	40.5
Medicaid	8.7	12.5	8.1	12.5	9.0	12.5
Private insurance	43.9	41.2	42.5	41.2	46.7	41.2
Self-pay	2.6	5.3	2.5	5.3	2.7	5.3
Other insurance	0.1	0.6	0.1	0.6	0.1	0.6
Hospital type						
Urban	82.7	84.1	81.3	84.1	85.2	84.1
Rural	17.3	15.9	18.7	15.9	14.8	15.9
Teaching status						
Non-teaching hospital	50.0	70.8	50.0	70.8		70.4
Teaching hospital	50.0	29.2	50.0	29.2		29.6
Rheumatoid diseases	2.3	4.7^a^	2.4	4.7^a^	1.9	4.7^a^
Congestive heart failure	5.1	5.5	6.2	5.5	2.6	5.5^a^
Chronic pulmonary disease	13.7	17.2^a^	13.7	17.2^a^	13.1	17.2^a^
Diabetes	14.2	15.4	13.6	15.4^a^	14.7	15.4
Hypertension	38.7	37.4	40.8	37.4^a^	33.3	37.4^a^
Hypothyroidism	8.9	10.2^a^	10.6	10.2	5.5	10.2^a^
Chronic Liver disease	4.5	4.0	5.6	4.0^a^	3.0	4.0
Obesity	7.0	8.2^a^	7.1	8.2	6.6	8.2
Chronic PE	2.8	1.7^a^	3.1	1.8^a^	1.9	1.8
Renal failure	7.3	8.2	7.2	8.2	7.7	8.2
Dyslipidemia	18.8	18.9	20.4	18.9	14.8	19.0^a^
Depression	9.9	15.1^a^	9.7	15.1^b^	11.3	15.1^a^
Psychoses	2.6	5.3^a^	2.6	5.3^b^	2.5	5.3^a^

Comorbidities independently associated with IBD-CRC

**Figure 1:**
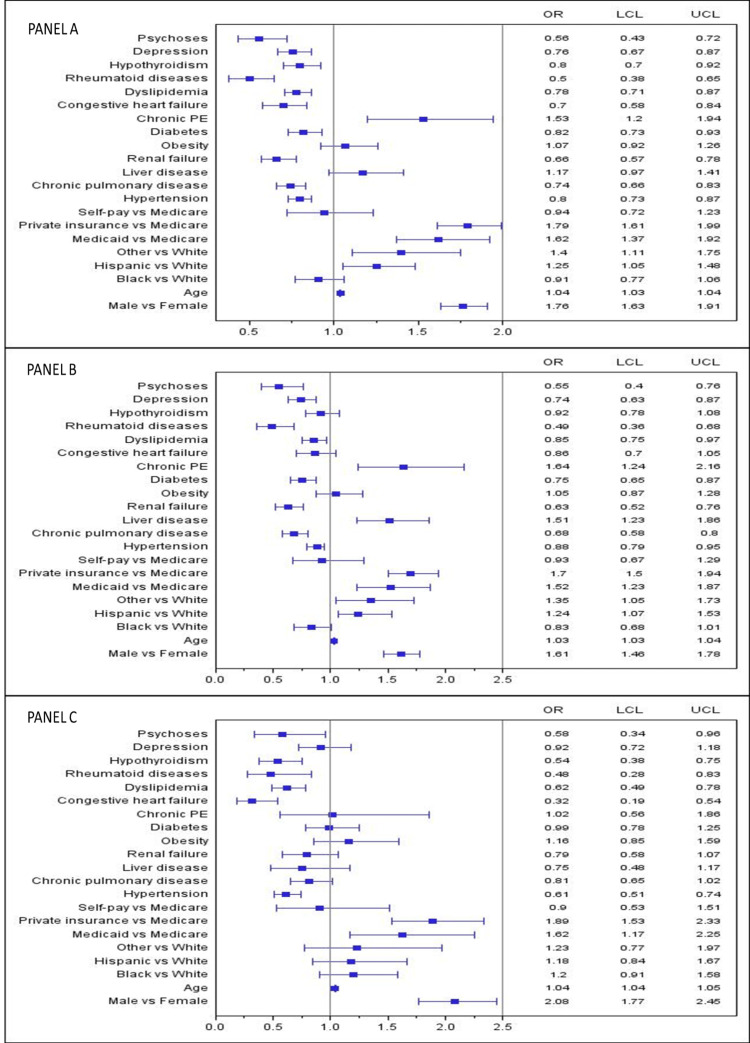
Factors associated with IBD-CRC, IBD-CC, IBD-RC patients Panel A: IBC-CRC findings, Panel B: IBD-CC findings, Panel C: IBD-RC findings, IBD: Inflammatory bowel disease, CRC: Colorectal cancer, CC: Colon cancer, RC: Rectal cancer, PE: Pulmonary embolism, OR: Adjusted odds ratio, LCL & UCL: Lower & upper-level confidence interval, Other race: includes Asian/Pacific Islanders, Native American, and mixed races

An increased risk of IBD-CC was seen in males (60%) of older ages and in those who had liver disease (OR 1.51, CI 1.23-1.86) and chronic PE (OR 1.64, CI 1.24-2.16). Also, patients <50 years old with liver disease were at an increased risk of IBD-CC in comparison to older patients (OR 1.83 vs 1.34, p<0.05). Although being obese was associated with an increased risk of IBD-CRC (OR 1.07, CI 0.92-1.26), IBD-CC (OR 1.05, CI 0.87-128), and IBD-RC (OR 1.16, CI 0.85-1.59), these associations were not statistically significant. Interestingly, hypertension, dyslipidemia, rheumatoid diseases, and psychoses were all inversely associated with the risk of IBD-CRC, IBD-CC, and IBD-RC in hospitalized patients (p<0.05) as shown in Figure 1, panels A, B, and C.

Comorbidities associated with IBD-CRC patients stratified by ulcerative colitis and Crohn’s disease

**Figure 2:**
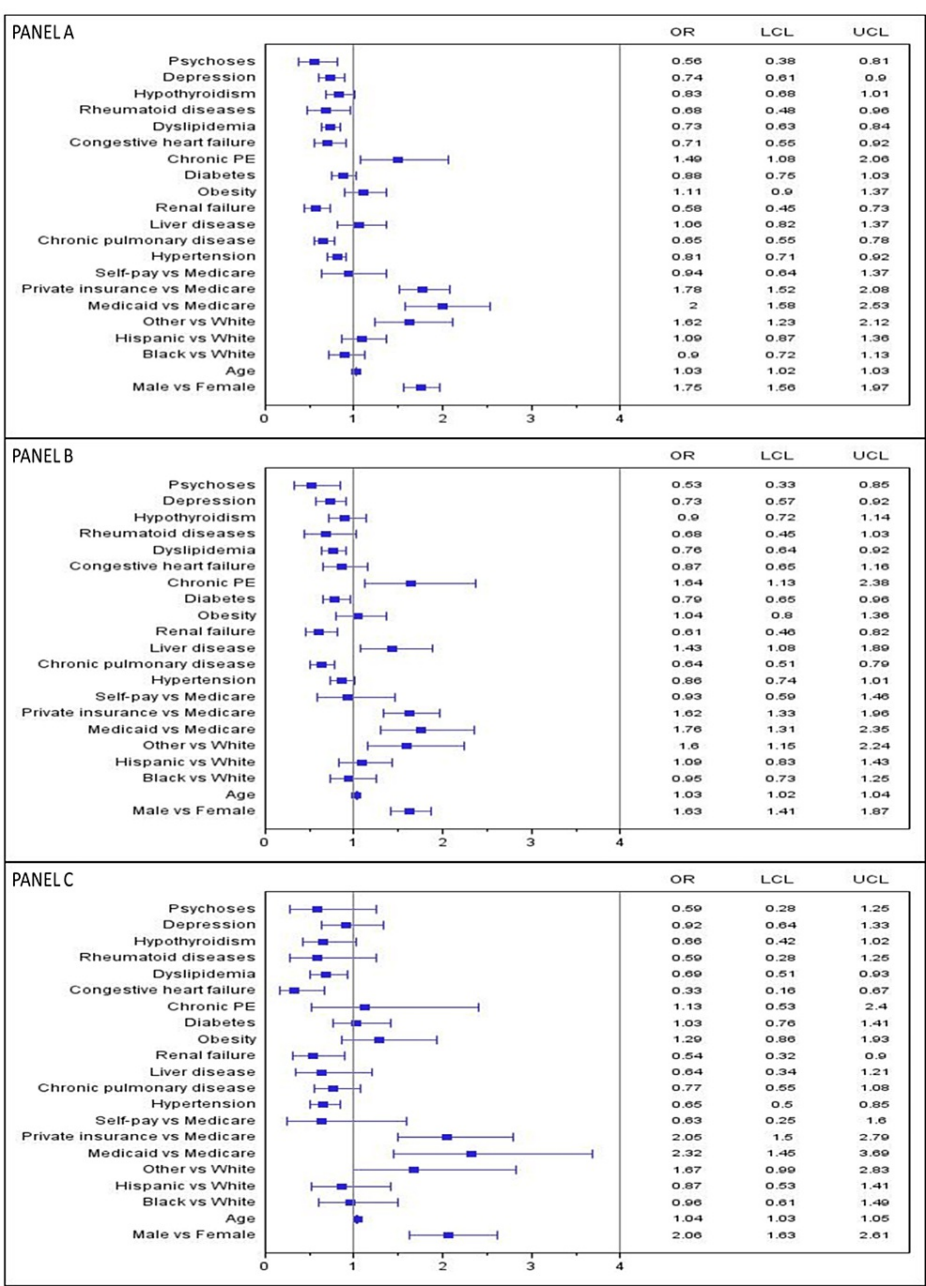
Factors associated with IBD-CRC, IBD-CC, and IBD-RC patients stratified to ulcerative colitis Panel A: IBC-CRC findings, Panel B: IBD-CC findings, Panel C: IBD-RC findings, IBD: Inflammatory bowel disease, CRC: Colorectal cancer, CC: Colon cancer, RC: Rectal cancer, PE: Pulmonary embolism, OR: Adjusted odds ratio, LCL & UCL: Lower & upper-level confidence interval, Other race: Includes Asian/Pacific Islanders, Native American, and mixed races

**Figure 3:**
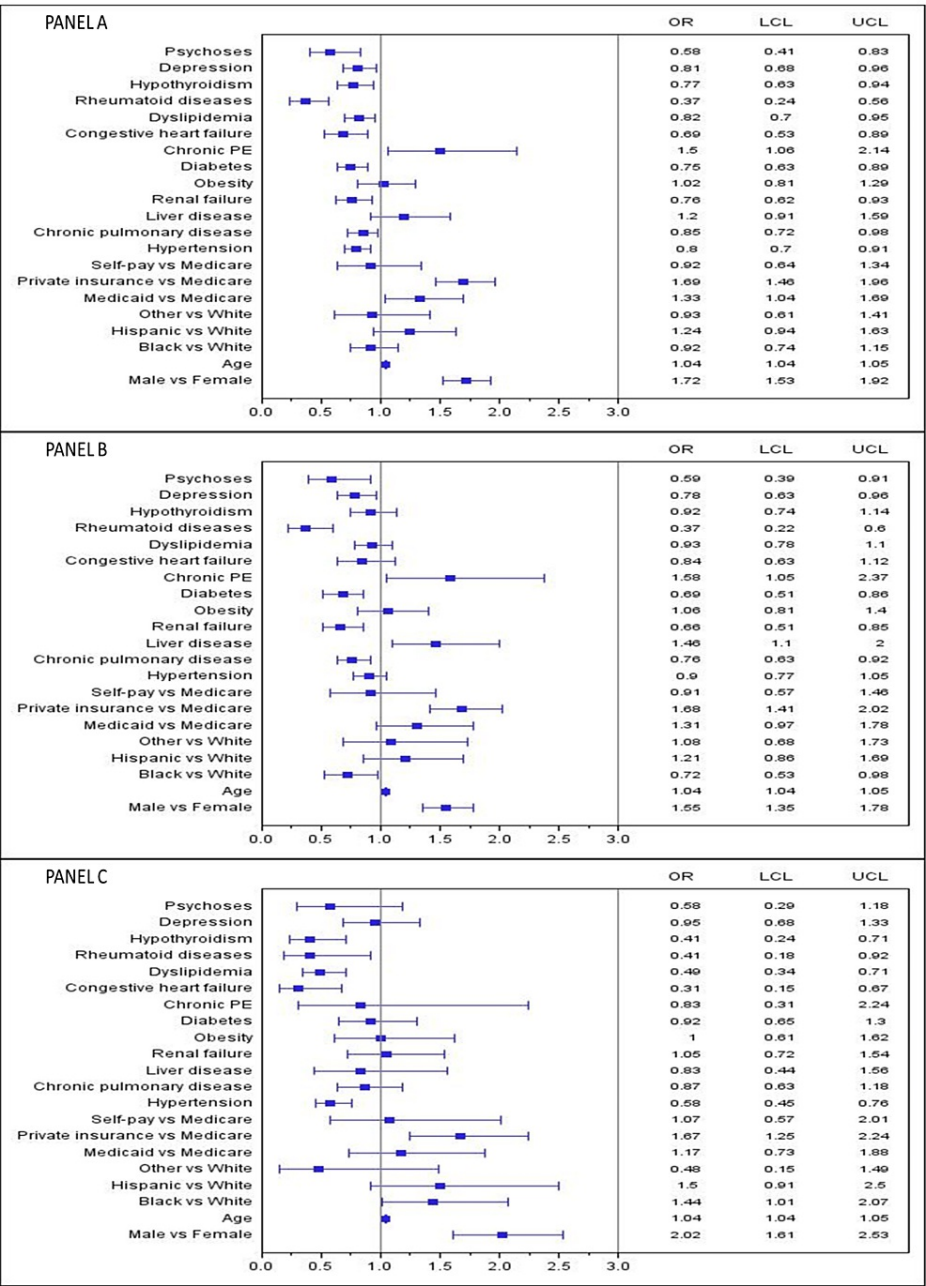
Factors associated with IBD-CRC, IBD-CC, and IBD-RC patients stratified to Crohn's disease Panel A: IBC-CRC findings, Panel B: IBD-CC findings, Panel C: IBD-RC findings, IBD: Inflammatory bowel disease, CRC: Colorectal cancer, CC: Colon cancer, RC: Rectal cancer, PE: Pulmonary embolism, OR: Adjusted odds ratio, LCL & UCL: Lower & upper-level confidence interval, Other race: Includes Asian/Pacific Islanders, Native American, and mixed races

After stratifying data by type of IBD, male sex, age, chronic PE (in IBD-CRC and IBD-CC), and liver disease (in IBD-CC) were still independently associated with an increased risk of IBD-associated cancer in both UC and CD patients (p<0.05). Renal failure and dyslipidemia were inversely associated with IBD-CRC, IBD-CC, and IBD-RC risk only in UC patients (p<0.05). Likewise, rheumatoid diseases were significantly associated with a lower risk of IBD-CRC, IBD-CC, and IBD-RC only in CD patients. Chronic pulmonary disease, depression, and psychoses were associated with a lower risk of IBD-CRC and IBD-CC but not IBD-RC in both IBD subgroups (p<0.05). Hypertension and congestive heart failure were associated with a lower risk of IBD-RC but not IBD-CRC and IBD-CC in UC and CD subgroups (Figure 2 and Figure 3; panels A, B, and C).

## Discussion

Association between liver disease, pulmonary circulation disorders, obesity, and IBD-CRC

To the best of the authors' knowledge, this is the first nationwide study in the US to assess the impact of comorbidities on the risk of IBD-related colon and rectal cancers. This study found that patients with IBD and liver diseases had an increased risk of colon cancer. The risk was higher in younger patients compared to the older population (age >50). Liver disease was also associated with an increased risk of IBD-CRC, although this association was not statistically significant. Likewise, having a chronic PE was associated with an increased risk of IBD-CRC, and IBD-CC. Patients with liver disease and chronic PE who have IBD thus may warrant closer CRC surveillance. Additionally, routine counseling on risk modification in these patients, such as smoking cessation, should be emphasized.

Patients with cholangitis and cholestatic disease were excluded from this study because IBD-CRC risk is well-established in diseases such as PSC [2,3]. Yet, the mechanism linking chronic non-cholestatic liver disease and IBD-CC was not clear. It has been suggested that the altered bile metabolism and inflammation associated with chronic liver disease might compound the proinflammatory state of IBD to increase the risk of IBD-CC, especially for the right-sided neoplasms [3,8]. Current guidelines recommend screening for colorectal cancer within one year of PSC diagnosis in a patient with IBD [3]. However, no guidelines exist currently to recommend CRC screening for IBD patients with chronic liver diseases such as hepatitis B or C and non-alcoholic steatohepatitis. Our findings suggest further research into this matter might be warranted.

Chronic PE in this study mostly represented PE with or without pulmonary infarcts and pulmonary hypertension. The risk of venous thromboembolism (VTE) in IBD patients is approximately three times that of the general population; the risk is also higher in hospitalized patients and patients with active disease [9]. Venous thromboembolism is more frequently seen in patients with active UC than in CD [10]. In our study, the percentage of hospitalized patients with chronic PE was higher in UC than in CD patients (2.2% vs 1.5%). As deep vein thrombosis (DVT)/PEs are frequently associated with malignancy, it is not surprising to find that chronic PE was positively associated with IBD-CRC in our study.

Although obesity is strongly associated with sporadic CRC [11], the link between obesity and IBD-associated CRC is largely unknown. Obesity appeared to be consistently associated with an increased risk of IBD-CRC in our study, however, these findings were not statistically significant. Similar to our study, Wu et al. (2019) who studied Chinese patients between 1999 and 2016 from eight tertiary-care hospitals noted no association between body mass index (BMI) and colonic neoplasia in IBD patients [12].

Association between diabetes, chronic pulmonary disease, renal failure, rheumatoid diseases, and IBD-CRC

Diabetes in our study was associated with a lowered risk of IBD-CRC by 18%. Suggested theories for this observation include the following: 1) common comorbidities such as diabetes, hyperlipidemia, hypertension, and obesity are less common among patients with IBD [13]; 2) metformin, a widespread medication in diabetes management has been shown to decrease the incidence of CRC by interfering with CRC cell growth, proliferation, and angiogenesis leading to cell death [14]; 3) chronic lung diseases are frequently encountered with IBD. It has been postulated that certain medications used to treat IBD including sulfasalazine, 5-aminosalicylic acid, methotrexate, azathioprine, and infliximab can induce lung disease [15]. Inflammatory bowel disease increases the risk of all-cause mortality in patients with chronic lung diseases and these patients may not live long enough to develop CRC [16]. This may explain why fewer IBD patients with chronic pulmonary disease had CRC in our study.

Renal failure is rare in IBD patients and is often classified as an extraintestinal manifestation [17]. In agreement with our data, the prevalence of renal involvement in IBD is between 4% to 23% [17]. The decreased associated risk of IBD-CRC in renal failure patients in our study could be the result of increased early mortality in this patient population due to end-stage renal disease (ESRD) [18].

Rheumatoid diseases were inversely associated with the risk of IBD-CRC. This is consistent with previous studies [19]. One possible explanation for this observation is the increased use of cyclooxygenase-2 (COX-2)-selective inhibitors in this group of patients. These medications have consistently been associated with a decreased risk of CRC in the meta-analysis of observational and randomized controlled trials [20]. Thus, it is no surprise that such a protective effect on the risk of CRC would extend to IBD patients with rheumatoid diseases.

Association between psychiatric disorders and IBD-CRC

The prevalence of psychiatric disorders (psychoses and depression) in IBD-CRC patients in our study was comparable to other published data [21-23] and was associated with a lower risk of IBD-CRC. Patients with IBD are known to have a high prevalence of mental illness leading to an increased risk of clinical relapse, disease severity, and surgery [22]. We suggest the following reasons for our findings: 1) patients with schizophrenia have been shown to express tumor suppressor genes and enhanced internal natural killer cell activities [23]; 2) patients with psychiatric illnesses might have an underestimated CRC rate due to less compliance to CRC screening and prevention procedures such as colonoscopies [23]; 3) many patients with depression are placed on selective serotonin reuptake inhibitors [24], which have been shown to reduce the risk of CRC; 4) patients with more severe IBD are most likely to develop depression and receive bowel resection such as colectomy if their diseases involve the colon. Through colectomy, their risk of developing CRC is significantly decreased [22].

Association between hypertension, dyslipidemia, and IBD-CRC

In our study, patients with IBD and hypertension had a lower risk of IBD-CRC. This observation may be related to the use of angiotensin-converting enzyme inhibitors (ACEIs) or angiotensin receptor blockers (ARBs) in hypertensive patients. In effect, the use of ACEIs/ARBs is near ubiquitous in hypertensive patients and has been inversely associated with CRC risk due to their antifibrotic properties [25].

Likewise, dyslipidemia is also associated with a lower risk for IBD-CRC. Patients with IBD have shown low levels of low-density lipoprotein cholesterol (LDL) compared to healthy subjects [13]; possibly due to statin use which has also been shown to reduce the risk of CRC in IBD patients [26].

Association between hypothyroidism, congestive heart failure, and IBD-CRC

We found an inverse association between hypothyroidism and IBD-CRC and IBD-RC patients. Similar to our study, L’Heureux et al. (2019) in Taiwan reported a 22% decrease in the risk association between hypothyroidism and CRC using a nationwide population-based cohort of 139,426 patients between 2008 to 2013 [27]. Thyroid hormone binding of cell surface receptor alpha-v-beta-3 (αVβ3) has been shown to lead to tumor cell proliferation and angiogenesis, and promotes viability in CRC cell lines [28]. Hence low levels of free thyroxine (T4) in hypothyroidism may protect against CRC by decreased interactions with these receptors.

Also, congestive heart failure in our study was inversely associated with the risk of IBD-CRC in both UC and CD patients. A possible explanation for this finding is that medications these patients often take including ACEIs/ARBs and statins may lower their cancer risk [25,26]. Our findings are contrary to those of Selvaraj et al. (2018) who did not find an association between heart failure and CRC in IBD patients [29]. However, their study was limited to men, and heart failure was determined by self-reporting without echocardiographic evidence.

Clinical implications

The findings of the current study have important clinical implications. To the best of our knowledge, few studies to date have evaluated factors associated with IBD-CRC using a comprehensive list of comorbidities. Comorbid conditions cannot be overlooked in patients with IBD because their presence may significantly change medical practice strategies as well as the patient’s prognosis [30]. This study may serve as a reference point for future studies to validate the impact of comorbidities on CRC risk in IBD patients at the population level. Also, colon cancer surveillance is both expensive and labor-intensive, and studies like ours done at the population level may enable practitioners to properly allocate resources to those IBD patients who have higher risk comorbidities.

Strengths and limitations

This study used a nationwide database with a large sample size of patients seen over five years. Our study reflected real-world medical practice as we included multiple common comorbidities encountered in clinical practice. Our study did have some limitations. The NIS data is an inpatient-only database, and hence chronic medical conditions and procedures encountered mostly in the outpatient setting might be underrepresented. Also, as the NIS data does not contain information on disease duration, severity, as well as medication used by patients, their associations with CRC in IBD patients cannot be ascertained.

## Conclusions

Using a large, nationwide database, we found a positive association between non-cholestatic chronic liver disease and colon cancer IBD patients. The risk of colon cancer persisted in young IBD patients with non-cholestatic liver disease. On the other hand, IBD patients with comorbidities such as diabetes, chronic pulmonary diseases, hypertension, renal failure, psychiatric illnesses, dyslipidemia, and rheumatic diseases may have a negative association with CRC. Population based studies are required to further investigate the role of these comorbidities in IBD-CRC risk.
